# Induction of histone deacetylases (HDACs) in human abdominal aortic aneurysm: therapeutic potential of HDAC inhibitors

**DOI:** 10.1242/dmm.024513

**Published:** 2016-05-01

**Authors:** María Galán, Saray Varona, Mar Orriols, José Antonio Rodríguez, Silvia Aguiló, Jaume Dilmé, Mercedes Camacho, José Martínez-González, Cristina Rodriguez

**Affiliations:** 1 Centro de Investigación Cardiovascular (CSIC-ICCC), Instituto de Investigación Biomédica (IIB-Sant Pau), 08025 Barcelona, Spain; 2 Laboratory of Atherothrombosis, Program of Cardiovascular Diseases, Center for Applied Medical Research, University of Navarra, 31008 Pamplona, Spain; 3 Laboratorio de Angiología, Biología Vascular e Inflamación y Servicio de Cirugía Vascular, Instituto de Investigación Biomédica (IIB-Sant Pau), 08025 Barcelona, Spain

**Keywords:** Abdominal aortic aneurysm, Histone deacetylases, Inflammation, Vascular remodelling, Metalloproteinases

## Abstract

Clinical management of abdominal aortic aneurysm (AAA) is currently limited to elective surgical repair because an effective pharmacotherapy is still awaited. Inhibition of histone deacetylase (HDAC) activity could be a promising therapeutic option in cardiovascular diseases. We aimed to characterise HDAC expression in human AAA and to evaluate the therapeutic potential of class I and IIa HDAC inhibitors in the AAA model of angiotensin II (Ang II)-infused apolipoprotein-E-deficient (*ApoE*^−/−^) mice. Real-time PCR, western blot and immunohistochemistry evidenced an increased expression of HDACs 1, 2 (both class I), 4 and 7 (both class IIa) in abdominal aorta samples from patients undergoing AAA open repair (*n*=22) compared with those from donors (*n*=14). Aortic aneurysms from Ang-II-infused *ApoE*^−/−^ mice exhibited a similar HDAC expression profile. In these animals, treatment with a class I HDAC inhibitor (MS-275) or a class IIa inhibitor (MC-1568) improved survival, reduced the incidence and severity of AAA and limited aneurysmal expansion evaluated by Doppler ultrasonography. These beneficial effects were more potent in MC-1568-treated mice. The disorganisation of elastin and collagen fibres and lymphocyte and macrophage infiltration were effectively reduced by both inhibitors. Additionally, HDAC inhibition attenuated the exacerbated expression of pro-inflammatory markers and the increase in metalloproteinase-2 and -9 activity induced by Ang II in this model. Therefore, our data evidence that HDAC expression is deregulated in human AAA and that class-selective HDAC inhibitors limit aneurysm expansion in an AAA mouse model. New-generation HDAC inhibitors represent a promising therapeutic approach to overcome human aneurysm progression.

## Abstract (precis)

**Summary:** This study reports the upregulation of HDACs in human AAA, evidences that HDAC inhibitors limit aneurysm progression in a preclinical model and suggests the therapeutic interest of HDAC inhibition in AAA.

## INTRODUCTION

Abdominal aortic aneurysm (AAA) is a degenerative vascular disease with high morbidity and mortality, and is common in elderly people. This disease affects up to 8-10% of men over the age of 65 years and its prevalence is predicted to increase in parallel with the global ageing population (Nordon et al., 2011; Weintraub, 2009). AAA rupture is the most severe consequence of this disease, which is among the 15 leading causes of death for people aged 60-84 years in the USA (Weintraub, 2009). Commonly, this is an asymptomatic disease characterised by an irreversible degradation of the vascular wall that results in tissue failure and progressive aortic dilatation. Surgical repair is currently the only therapeutic strategy for AAA, but is associated with a substantial perioperative risk (Powell and Brady, 2004).

AAA is characterised by chronic inflammation of the aortic wall that is associated with upregulation of metalloproteinases (MMPs), depletion of vascular smooth muscle cells (VSMCs) by apoptosis and enhanced neovascularisation (Golledge et al., 2006; Forsdahl et al., 2009; Camacho et al., 2013). The mechanisms underlying AAA expansion and rupture are complex and a better understanding of the processes involved in AAA progression is crucial for the development of new therapeutic strategies. Although numerous pharmacological interventions have been proposed to limit AAA growth, none of them have provided convincing results in clinical trials (Samson, 2012; Shiraya et al., 2009; Dodd and Spence, 2011).

Recent evidence highlights the importance of epigenetics in the development of cardiovascular diseases. Among epigenetic mechanisms, those governed by histone deacetylases (HDACs) strongly affect gene transcription (Narlikar et al., 2002; Hildmann et al., 2007). HDACs constitute a family of 18 molecules divided into four classes [I, II (comprising class IIa and IIb), III, IV]. Class I and IIa HDACs regulate the expression of genes involved in key events in AAA, including VSMC differentiation, contractility, proliferation, inflammation and extracellular matrix deposition (Pons et al., 2009). Interestingly, HDAC inhibitors have been proven effective in several types of cancer (Leoni et al., 2002; Bolden et al., 2006; Minucci and Pelicci, 2006) and represent a promising therapy for non-oncological diseases, including neurodegeneration, inflammation and cardiovascular diseases (Pons et al., 2009; Colussi et al., 2010). However, the relevance of the different HDACs in human AAA has not been established. Based on the aforementioned data, we hypothesised that HDACs could be crucial in human AAA and that novel class-selective HDAC inhibitors could bring new therapeutic opportunities for AAA.

## RESULTS

### Class I and II HDACs are augmented in human AAA and in the Ang-II-infused *ApoE*^−/−^ mouse model

**Fig. 1.**
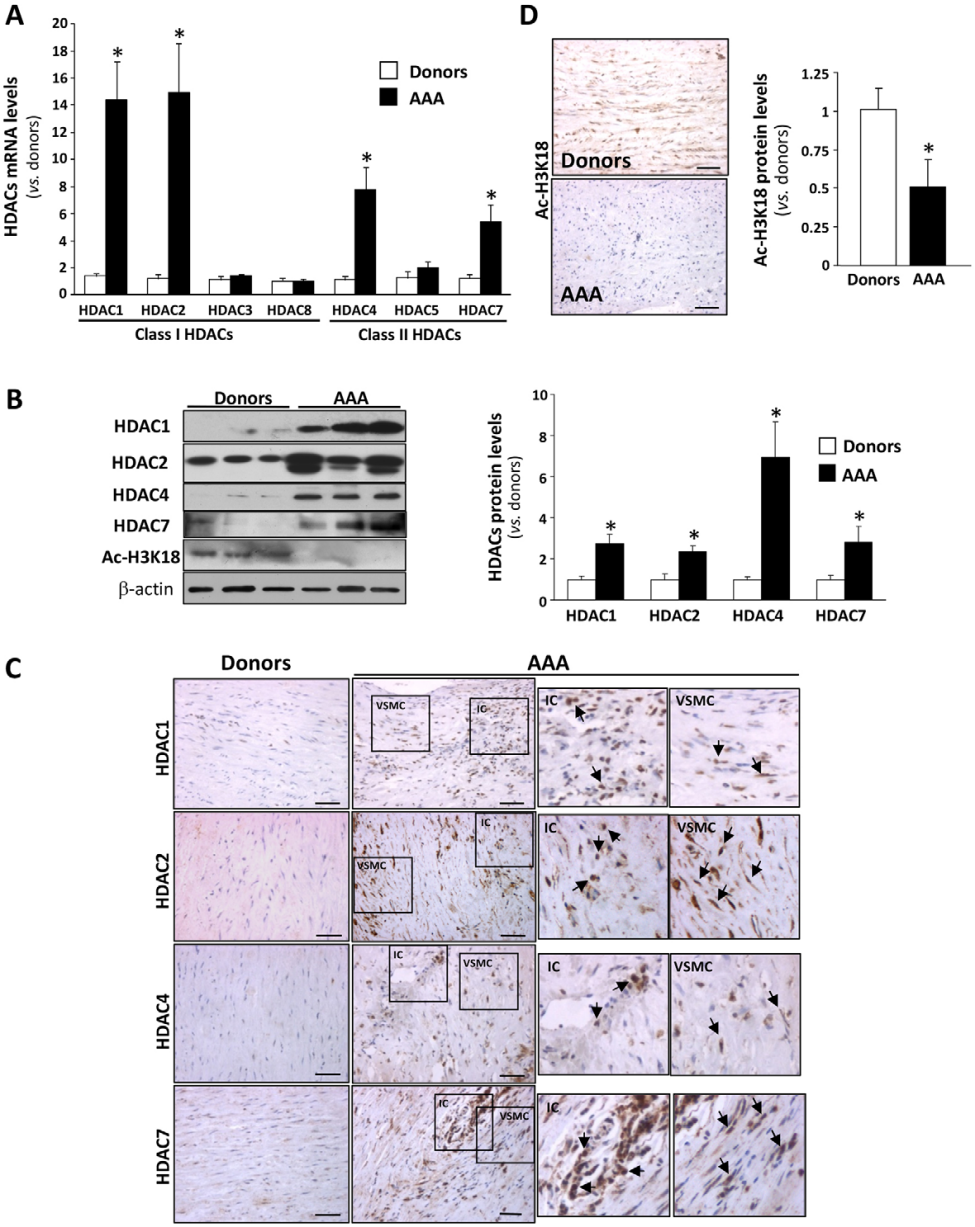
**Class I and II HDACs are upregulated in human abdominal aortic aneurysm (AAA).** (A) Class I and IIa HDAC mRNA levels analysed by quantitative real-time PCR in abdominal aorta from AAA patients and donors (controls). Values shown are the mean±s.e.m. (AAA: *n*=22; donors: *n*=14). (B) Representative western blot analysis of HDAC1, 2, 4 and 7 (left panel) and their corresponding quantitative histogram (right panel; AAA: *n*=1; donors: *n*=8). (C) Representative immunostaining for HDAC1, 2, 4 and 7 in haematoxylin-counterstained aortic samples from AAA patients and controls. Scale bars: 50 μm. IC, inflammatory cells; VSMC, vascular smooth muscle cells. Arrows indicate positively stained cells. (D) Densitometric analysis of Ac-H3K18 protein levels analysed by western blot (shown in B) and immunohistochemistry in haematoxylin-counterstained aortic samples from AAA patients and controls. Results are expressed as mean±s.e.m. *P≤0.05 vs donors (*t*-test and one-way ANOVA).

The expression pattern of class I and class IIa HDACs was analysed in abdominal aorta samples from AAA patients (*n*=22) and donors (*n*=14). The abdominal aorta from AAA patients exhibit extreme structural abnormalities characterised by a destructive connective tissue remodelling and an important inflammatory infiltrate, as described previously (Camacho et al., 2013; Golledge et al., 2006). *HDAC1*, *2*, *4* and *7* mRNA levels were significantly upregulated in AAA (between 15- and 5-fold) compared with healthy controls, whereas no differences were observed for *HDAC3*, *5* and *8* (Fig. 1A). The upregulation of HDACs was confirmed by western blot (Fig. 1B) and was further assessed by immunohistochemistry. A strong immunostaining for HDAC1, 2, 4 and 7 was detected in the media layer of aneurysmal samples, specifically in VSMCs and in areas that were enriched with inflammatory cells (Fig. 1C). Double-immunofluorescence analysis in human AAA samples corroborate that HDAC1, 2 and 4 are expressed by both smooth muscle α-actin cells (VSMCs) and CD3-positive cells (T cells) (Fig. S1). Consistently, the enhanced expression of HDACs in AAA was associated with a decrease in the degree of histone H3 acetylation (Fig. 1B,D). Clinical data of patients and donors are shown in Table 1.

**Table 1.**
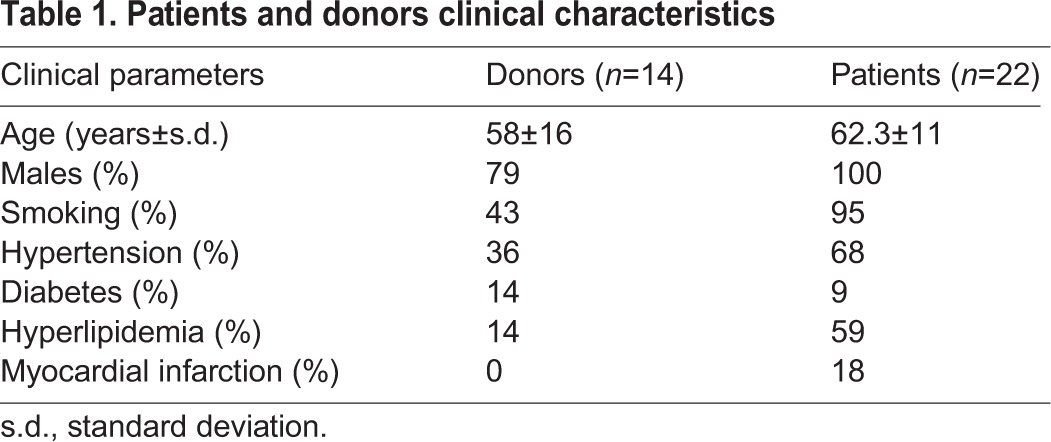
**Patients and donors clinical characteristics**

The Ang-II-infused *ApoE*^−/−^ mouse is a well-established model of AAA (Daugherty et al., 2000). Similarly to what is observed in humans, the expression of *HDAC1*, *2*, *4* and *7*, and also that of HDAC3, was increased in the aorta of *ApoE*^−/−^ mice after 4 weeks of Ang-II infusion, compared with saline-infused animals, whereas mRNA levels for *HDAC5* and *8* remained unchanged (Fig. S2).

### HDAC inhibitors reduced mortality rates in Ang-II-infused *ApoE*^−/−^ mice and limited the incidence and severity of AAA

Owing to the strong upregulation of HDACs in AAA, we aimed to determine whether HDAC inhibition could limit aneurysm development. Ang-II-infused *ApoE*^−/−^ mice were treated with two well-known class-selective HDAC inhibitors, MS-275 (an inhibitor of class I HDACs) and MC-1568 (a class IIa HDAC inhibitor) (Beckers et al., 2007; Fleming et al., 2014). Ang-II infusion for 4 weeks increased systolic blood pressure and mean arterial pressure, but neither affected lipid profile nor body weight, as previously described (Daugherty et al., 2000). None of these parameters was altered by the inhibition of HDAC activity (Table S1). Furthermore, both inhibitors markedly reduced the cardiac hypertrophic response to Ang II (data not shown), in agreement with previous data (Kee et al., 2006; Kong et al., 2006).

**Fig. 2.**
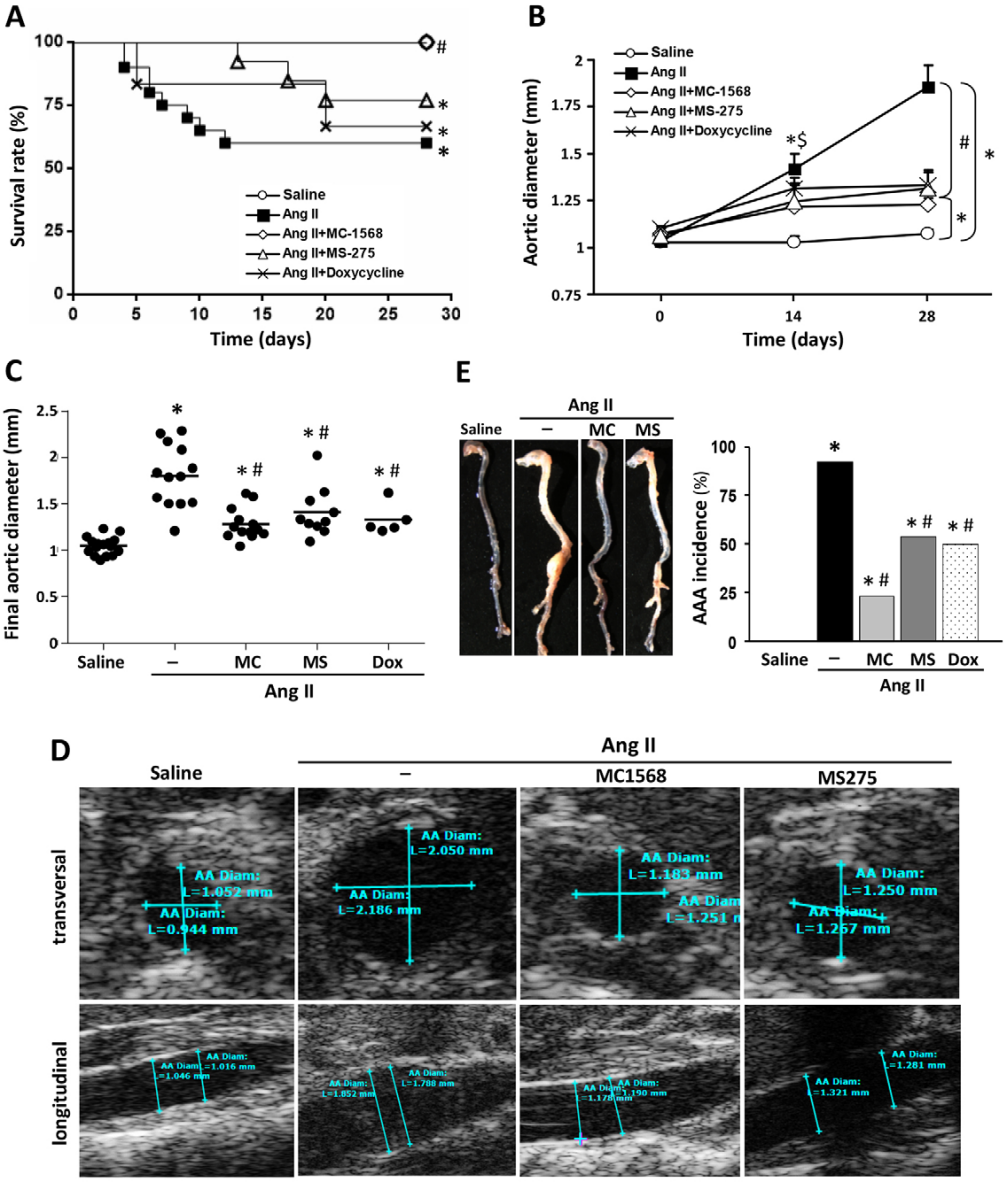
**The progression of abdominal aortic aneurysm (AAA) is limited by class I and class IIa HDAC inhibitors.** *ApoE*^−/−^ mice were infused with saline or Ang II and were treated or not with MC-1568 (MC), MS-275 (MS) or doxycycline (Dox). (A) Graph showing the survival rates of each group of animals. Please note that data from the MC-1568 group, represented by an opened diamond, overlap the open circle symbol corresponding to saline-infused animals. All animals from both saline and MC-1568 groups survived until the end of the study. *P<0.05 vs saline at 28 days; ^#^*P*<0.05 vs Ang II at 28 days (Kaplan–Meier analysis). (B) Time-course analysis of abdominal aortic diameters (mm) evaluated by ultrasonography at 0, 14 and 28 days of Ang II infusion in each group. Data are expressed as mean±s.e.m. *P<0.05 vs saline at 14 and 28 days; ^#^*P*<0.05 vs Ang II at 28 days; and ^$^*P*<0.05 Ang II vs Ang II+MC-1568 at 14 days (two-way ANOVA/Bonferroni test). (C) Maximal suprarenal abdominal aortic diameter (in mm) measured from transverse ultrasound images at day 28 post-infusion. Data are expressed as mean±s.e.m. *P<0.05 vs saline; ^#^*P*<0.05 vs Ang II (one-way ANOVA/Mann–Whitney test). (D) Representative high-frequency ultrasound frames of abdominal aortas from all groups. Transverse (top) and longitudinal (bottom) images were taken at the level of the suprarenal aorta. AA, abdominal aorta; L, length. (E) Representative images of fixed aortas. The histogram represents the incidence of AAA (including AAA-related mortality) in percentage in each group (*n*=10-13). *P<0.05 vs saline; ^#^*P*<0.05 vs Ang II (χ^2^ test).

Ang-II infusion resulted in the early death of some animals owing to aortic rupture, whereas HDAC inhibition delayed death episodes (Fig. 2A). The percentage of Ang-II-infused *ApoE*^−/−^ mice that survived over the course of the study was 62%, with deaths occurring after the fifth day post-infusion. MS-275 treatment did not significantly improve the survival rate, although this drug delayed fatal events by 1 week. Interestingly, all Ang-II-infused *ApoE*^−/−^ mice treated with MC-1568 survived until the end of the study (Fig. 2A). Ang-II-infused *ApoE*^−/−^ mice treated with doxycycline, a broad-spectrum MMP inhibitor that inhibits AAA development in this model (Manning et al., 2003), showed a 70% survival rate.

In order to follow-up AAA progression throughout the study, aortic diameter at 0, 14 and 28 days after Ang-II infusion was monitored and recorded by ultrasonography (Fig. 2B). Ang II progressively increased abdominal aortic diameter, whereas HDAC inhibitors significantly decreased aortic diameter 14 days after treatment and no further progression was observed (Fig. 2B). The MMP inhibitor doxycycline, used as a positive control, produced a similar effect. Data in Fig. 2C and ultrasound images at the end of the study (Fig. 2D) evidenced the significant attenuation of aortic diameter exerted by these drugs.

Ang-II infusion in *ApoE*^−/−^ mice induced the formation of suprarenal aneurysm and structural alterations in the whole aorta, whereas no spontaneous AAAs were observed in saline-infused mice (Fig. 2E, left panel). After Ang-II infusion, only 23% of mice treated with MC-1568 and 54% of those receiving MS-275 developed AAA, in contrast to the high incidence in *ApoE*^−/−^ mice (92%). The preventive effect of MS-275 on AAA incidence was similar to that exerted by doxycycline (50%; Fig. 2E, right panel). Control mice injected with vehicle [0.5% carboxymethylcellulose or 50% dimethyl sulfoxide (DMSO) alone] did not show alterations in aortic diameter or in aorta morphology (not shown). At the end of the procedure, Ang-II-infused mice exhibited the most severe forms of AAA (Table 2). The treatment with HDAC inhibitors reduced the severity of aneurysmal lesions. Mice from the MC-1568 group showed a low incidence of aneurysms and less severe forms of them, whereas the MS-275 group exhibited an intermediate phenotype.

**Table 2.**
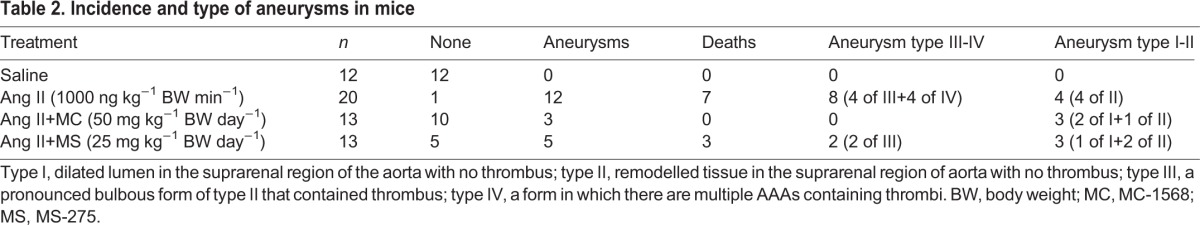
**Incidence and type of aneurysms in mice**

**Fig. 3.**
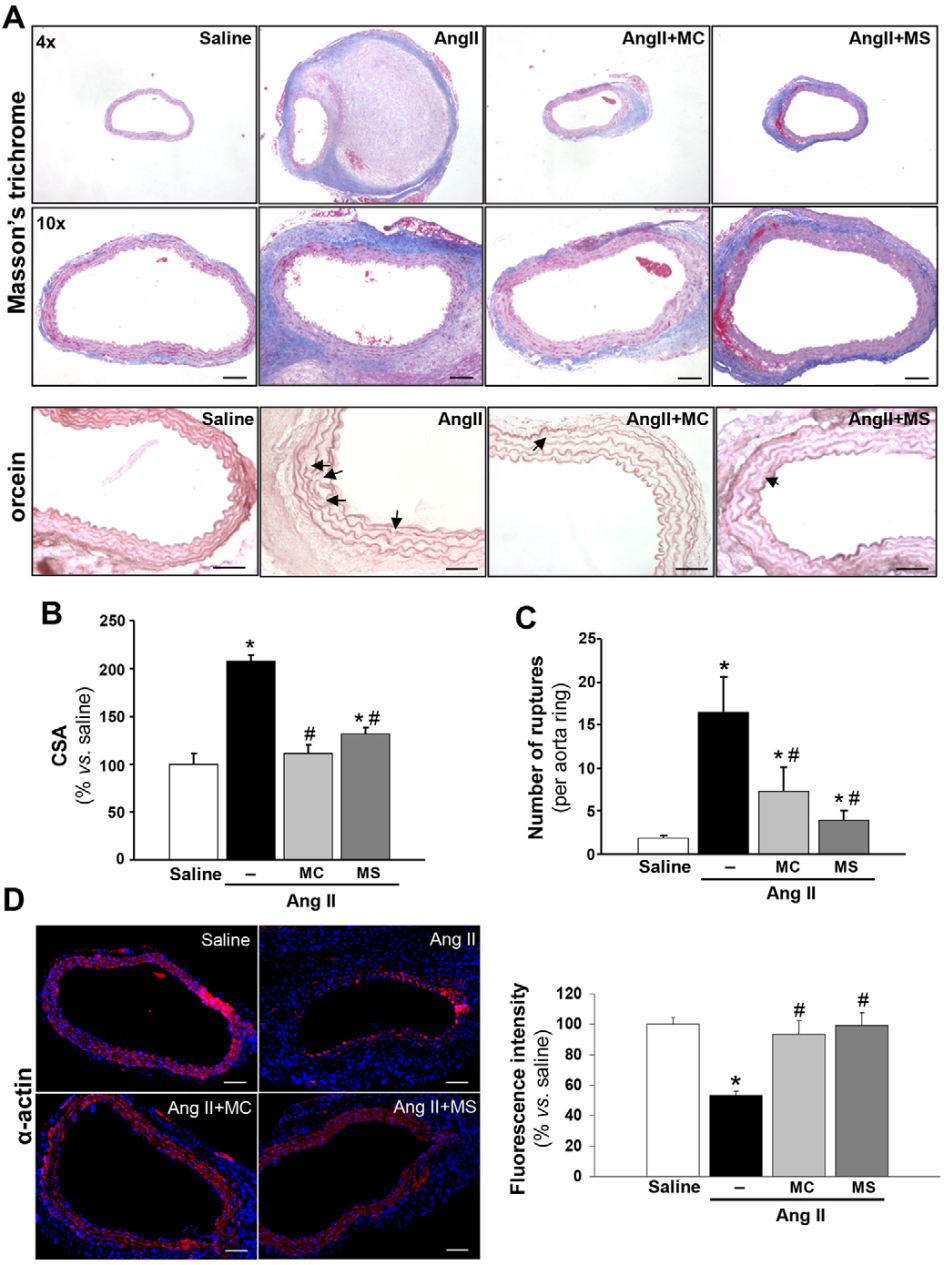
**Collagen deposition, wall thickness and elastin degradation are reduced by HDAC inhibitors in the abdominal aorta from Ang-II-infused *ApoE*^−/−^ mice.** *ApoE*^−/−^ mice were infused with saline or Ang II. Ang-II-infused mice were treated with MC-1568 (MC) or MS-275 (MS). (A) Representative aortic sections stained with Masson's trichrome captured with 4× and 10× objectives as indicated (upper panels; scale bars: 200 μm). Orcein staining showed in the lower panels allows the analysis of elastic fibre morphology (lower panels; scale bars: 50 μm). Arrows indicate elastic fibre ruptures. (B) Histogram showing cross-sectional area (CSA). (C) Quantification of the number of ruptures of elastin fibres. Results are expressed as mean±s.e.m. from *n*=5. *P<0.05 vs saline and ^#^*P*<0.05 vs Ang II (*t*-test and one-way ANOVA). (D) Representative immunofluorescence staining of α-actin (red) in aortic sections from all groups of mice. Nuclei were counterstained with DAPI (blue). Scale bars: 200 μm. Graph shows fluorescence intensity.

Histological examination of the suprarenal aorta showed that HDAC inhibitors attenuated the Ang-II-induced disturbance in overall abdominal aorta morphology. These drugs blocked the marked increase in aortic wall thickness and collagen deposition observed in Ang-II-infused *ApoE*^−/−^ mice [Fig. 3A (upper panels), B]. Additionally, orcein staining showed that inhibition of either class I or class II HDACs reduced the disruption of elastic lamina that was frequently observed in Ang-II-infused mice [Fig. 3A (lower panel), C]. Immunostaining for smooth muscle α-actin showed that VSMC density was remarkably reduced in the media of aneurysm sections from Ang-II-infused mice compared with controls. Interestingly, both HDAC inhibitors prevented such an effect (Fig. 3D). Apoptosis is a key factor that contributes to medial degeneration in AAA (Thompson et al., 1997). In agreement with the reduction in medial VSMC density observed in Ang-II-infused animals, we detected cleaved-caspase-3-positive cells in these mice, an effect that was prevented by MC-1568 or MS-275 (Fig. S3A), and similar results were obtained by TUNEL assay (Fig. S3B).

### HDAC inhibitors attenuate the Ang-II-induced systemic and vascular inflammatory response

**Fig. 4.**
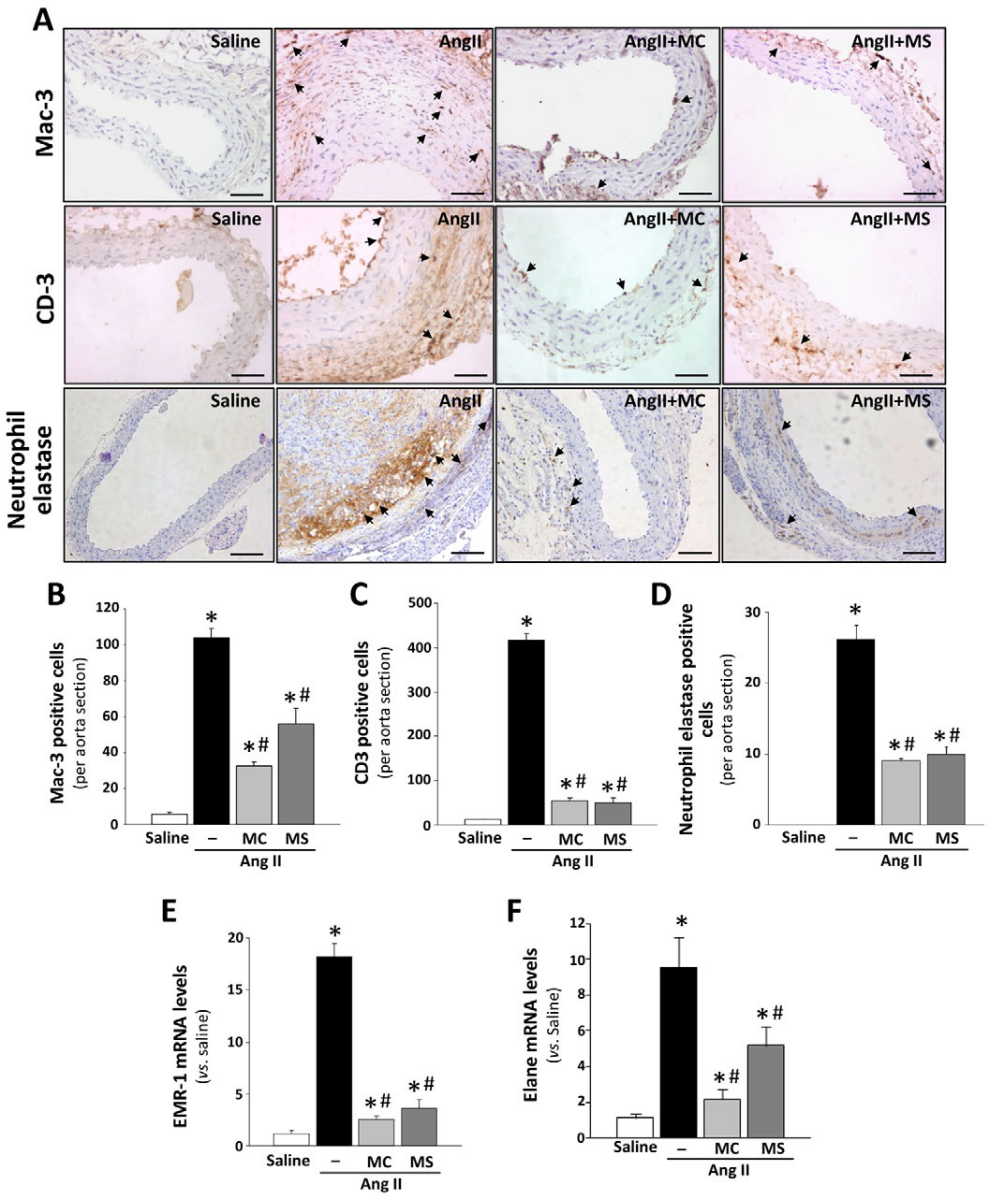
**Immune cell infiltration is reduced by HDAC inhibitors in abdominal aorta from Ang-II-infused** ***ApoE*^−/−^ mice.** *ApoE*^−/−^ mice were infused with saline or Ang II. Ang-II-infused mice were treated or not with MC-1568 (MC) or MS-275 (MS). (A) Representative images corresponding to macrophage (Mac-3, upper panels), lymphocyte (CD3, middle panels) and neutrophil [elastase, neutrophil expressed (Elane); lower panels] infiltration analysed by immunohistochemistry. Arrows indicate positive cells for each marker. Scale bars: 50 μm. (B-D) Histograms show the quantification of cells positive for Mac-3 (B), CD3 (C) and neutrophil elastase (D) per aortic section (*n*=5). (E,F) The aortic expression of *EMR-1*, a macrophage marker (E), and *Elane*, a neutrophil marker (F), were evaluated by real-time PCR and normalised to GAPDH expression (*n*=8). Values shown are mean±s.e.m. *P<0.05 vs saline; ^#^*P*<0.05 vs Ang II (*t*-test and one-way ANOVA).

**Fig. 5.**
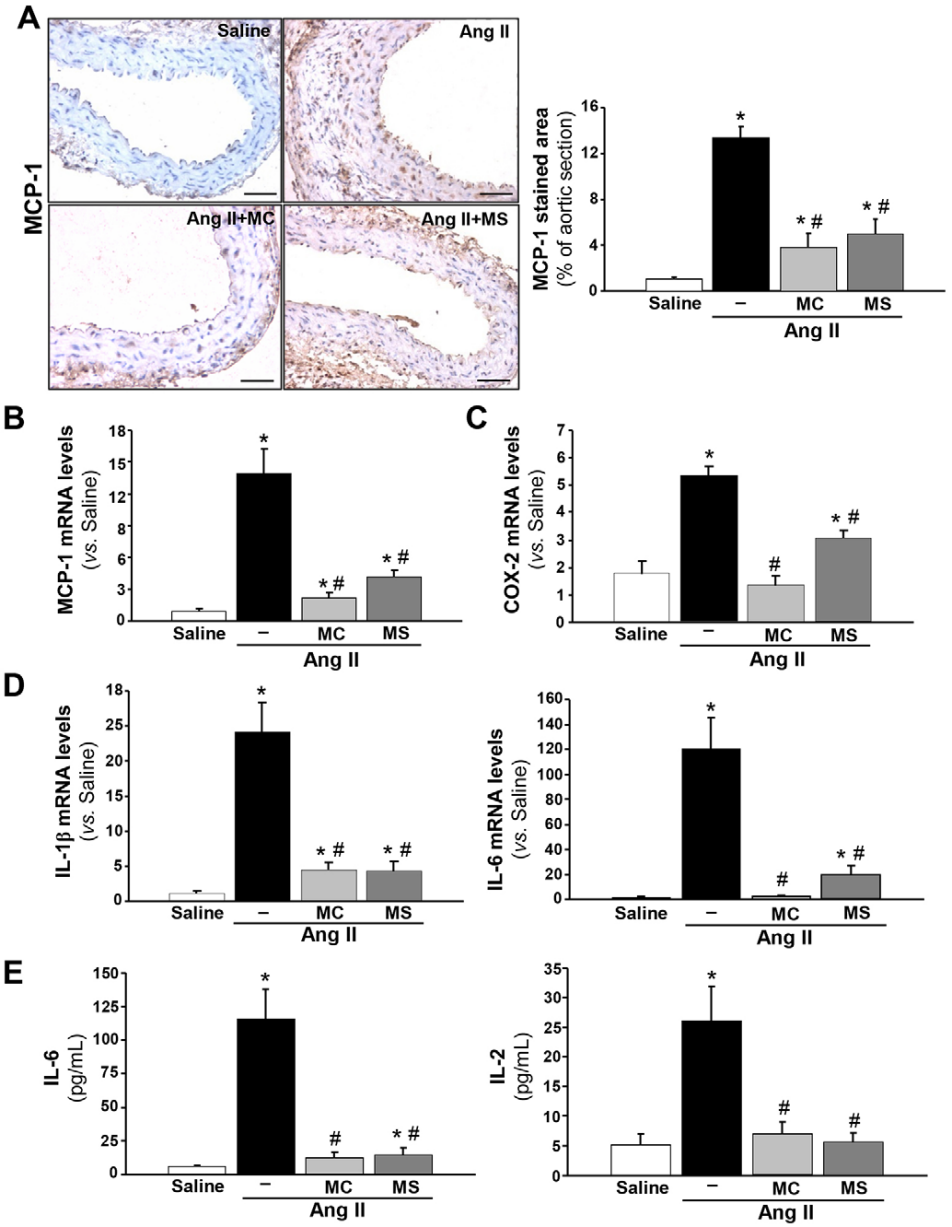
**HDAC inhibitors limit vascular expression of proinflammatory markers in the abdominal aorta from Ang-II-infused *ApoE*^−/−^ mice.** *ApoE*^−/−^ mice were infused with saline or Ang II. Ang-II-infused mice were treated or not with MC-1568 (MC) or MS-275 (MS). (A) Immunohistochemical analysis of MCP-1 (left panels) and quantitative analysis of the positive-stained area (right) (*n*=5; scale bars: 50 μm). (B-D) mRNA levels of *MCP-1* (B), *COX-2* (C) and interleukin (IL)-1β (D, left panel) and IL-6 (D, right panel) normalised to *GAPDH* (*n*=8). (E) IL-6 and IL-2 systemic levels (*n*=6). Data are the mean±s.e.m. *P<0.05 vs saline; ^#^*P*<0.05 vs Ang II (*t*-test and one-way ANOVA).

The activation of the inflammatory response is a well-established process in both human aneurysm and Ang II-induced experimental AAA (Freestone et al., 1995; Shimizu et al., 2006). In agreement, a marked increase in the accumulation of infiltrated macrophages, T-cells and neutrophils into the vessel wall of Ang-II-infused *ApoE*^−/−^ mice was revealed by immunohistochemistry, as compared to animals receiving saline (Fig. 4A). Mac3-, CD3- and neutrophil-elastase-positive cells were abundant in *ApoE*^−/−^ mice infused with Ang II but not in control aortas, whereas treatment with either HDAC inhibitor significantly reduced the immune infiltrate (Fig. 4B-D). Furthermore, the drastic upregulation in the expression of EMR-1 (a macrophage marker) and Elane (a neutrophil marker) induced by Ang II was attenuated by HDAC inhibition (Fig. 4E,F). Likewise, immunohistochemistry and real-time PCR analysis evidenced that both MC-1568 and MS-275 prevented the vascular increase in *MCP-1* (monocyte chemotactic protein-1) expression triggered by Ang II in *ApoE*^−/−^ mice (Fig. 5A,B) and, similarly, these drugs reduced the enhanced expression of other pro-inflammatory mediators, including cyclooxygenase 2 (COX-2; Fig. 5C) and interleukin (IL)-1β and -6 (Fig. 5D). Likewise, the striking increase in the circulating levels of IL-6 and IL-2 in *ApoE*^−/−^ mice in response to Ang II was significantly reduced by class I and II HDAC inhibition (Fig. 5E). HDAC inhibitors also attenuated the inflammatory response triggered by Ang II in human VSMCs in culture (Fig. S4A-C). These results suggest a direct implication of HDACs in the regulation of the inflammatory response in this AAA model.

### HDAC inhibitors decrease MMP expression/activity in Ang-II-infused *ApoE*^−/−^ mice

**Fig. 6.**
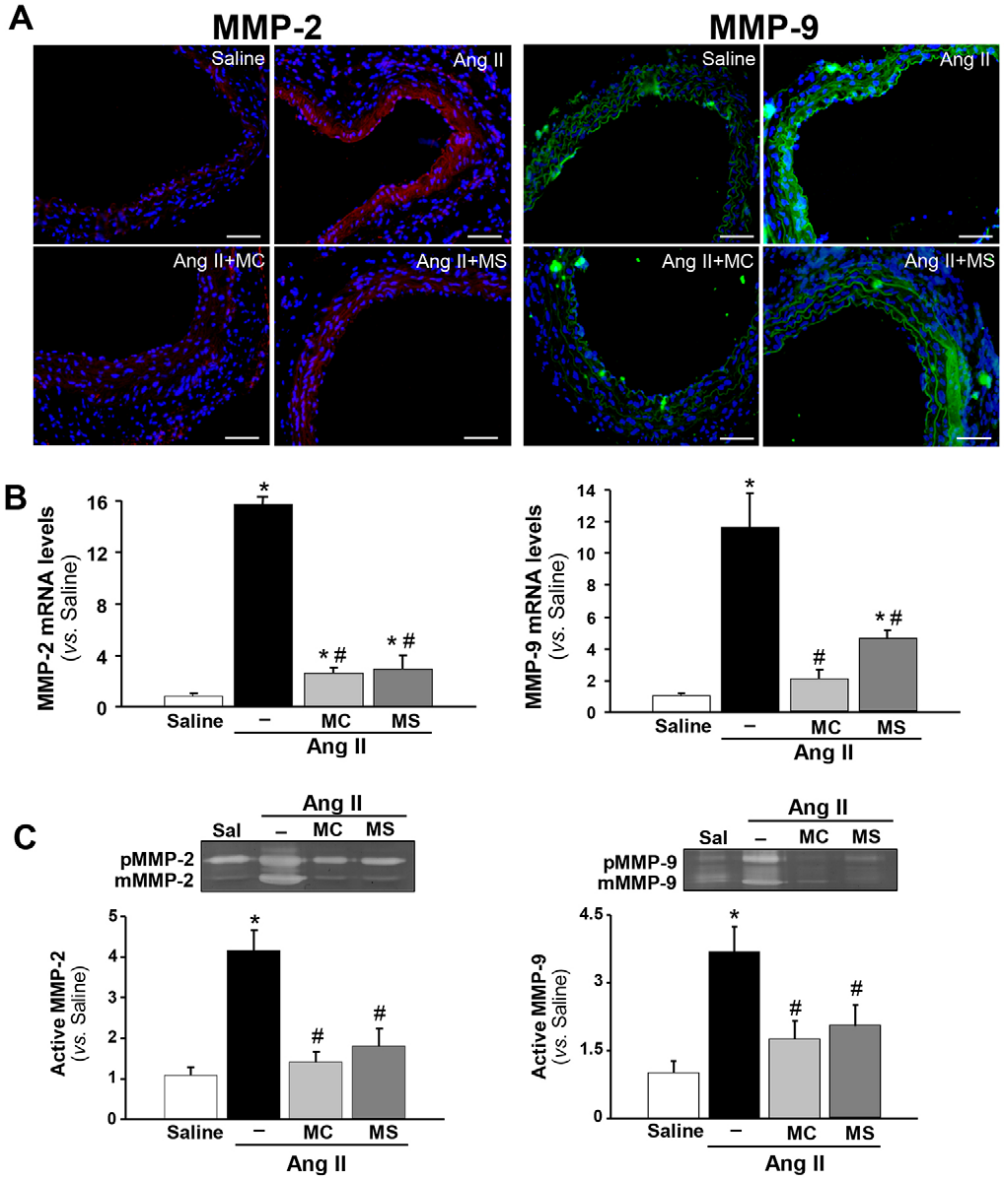
**HDAC inhibition reduced MMP-2 and -9 expression and activity in abdominal aorta from Ang-II-infused** ***ApoE*^−/−^ mice.** *ApoE*^−/−^ mice were infused with saline (Sal) or Ang II. Ang-II-infused mice were treated or not with MC-1568 (MC) or MS-275 (MS). (A) Representative images from abdominal aorta sections stained by immunofluorescence for MMP-2 (red, left panels) and MMP-9 (green, right panels). Nuclei were counterstained with DAPI (blue) (*n*=5; scale bars: 50 µm). (B) *MMP-2* (left panel) and *MMP-9* (right panel) mRNA levels analysed by real-time PCR and normalised to *GAPDH* (*n*=8). (C) Representative gelatin gel zymography to detect MMP zymogens (pMMP) and active forms (mMMP) in protein extracts of murine abdominal aortas from each group. Histograms represent the densitometric quantification of active forms for both MMPs (*n*=5). Results are shown as the mean±s.e.m. *P<0.05 vs saline; ^#^*P*<0.05 vs Ang II (*t*-test and one-way ANOVA).

We assessed the repercussion of HDAC inhibition on the proteolytic process mediated by MMPs in Ang-II-induced aneurysm. MMP-2 and MMP-9 are the main isoforms that degrade elastic fibres and collagen in mouse models of AAA (Davis et al., 1998; Pyo et al., 2000; Longo et al., 2002; Luttun et al., 2004). As expected, a marked induction of MMP-2 and MMP-9 expression was detected in the abdominal aorta from Ang-II-infused *ApoE*^−/−^ mice, as evidenced by immunofluorescence (Fig. 6A) and real-time PCR analysis (Fig. 6B). This increase was significantly prevented by both MC-1568 and MS-275 (Fig. 6A,B). In accordance, zymography assays performed with abdominal aorta lysates demonstrate that Ang II induced the zymogens and active forms of MMP-2 and MMP-9 (Fig. 6C). Interestingly, class I and class IIa HDAC inhibitors elicited a significant attenuation in the activity of these MMPs and a reduction in their pro-form levels (Fig. 6C). Similarly, these drugs limited the induction of MMP-2 expression evoked by Ang II in human VSMCs in culture (Fig. S4D).

## DISCUSSION

Therapeutic strategies in AAA are restricted to invasive surgical repair of those AAAs under a high risk of rupture and, unfortunately, there are still no effective pharmacological drugs that limit AAA progression and rupture. Although statins, doxycycline, vitamin E, COX-2 inhibitors, angiotensin-converting enzyme inhibitors and Ang-II-receptor blockers have been suggested to reduce AAA expansion (Golledge et al., 2006; Samson, 2012; Dodd and Spence, 2011; Manning et al., 2003), none of these therapies has conclusively demonstrated their clinical benefit. Therefore, the search for new therapeutic targets in AAA is a challenge that requires a deep understanding of the mechanisms involved in this disease.

In recent years, an increasing number of studies have reported that an exacerbated expression/activity of HDACs could be detrimental in multiple pathologies, including cardiovascular diseases (Pons et al., 2009; Trivedi et al., 2007; Findeisen et al., 2011). Despite the outstanding progress in the knowledge of the pathophysiological role of HDACs in cardiovascular pathologies, little is known about their involvement in AAA formation, expansion and rupture. Our results evidence for the first time the induction of HDACs in human AAA. Furthermore, in an experimental model of Ang-II-induced AAA, class I and class IIa HDAC inhibitors largely reduced AAA progression, suggesting that HDACs could play an important role in aneurysm formation.

In human aneurysmal samples, specific HDACs from class I and II were induced, in particular HDAC1, 2 (both class I), 4 and 7 (both class IIa), supporting their involvement in the human pathology. This induction was observed in VSMCs and mainly in the inflammatory infiltrate. In accordance, we observed a reduction in the degree of histone H3 acetylation in human AAA most likely secondary to the induction of HDAC expression. In contrast, an increased histone H3 acetylation has been reported in VSMCs in culture from patients with Marfan and non-Marfan thoracic aneurysm (TAA) related to a slight enhancement in histone acetyltransferase activity (Gomez et al., 2011). Hence, these divergent epigenetic mechanisms that seem to participate in AAA and TAA could explain the different etiopathogenesis of these diseases.

Interestingly, a similar expression pattern of class I and class II HDACs was found in the abdominal aorta of *ApoE*^−/−^ mice infused with Ang II and, hence, the HDAC-dependent changes detected in this model resemble those observed in human disease. Owing to the reversibility of histone acetylation, HDAC inhibition raised as an attractive therapeutic option. Our results demonstrate that systemic administration of selective class I and class IIa HDAC inhibitors in this model delayed mortality and limited AAA expansion and severity. An early study reported that Metacept, a highly cytotoxic class I HDAC inhibitor (Shehu-Xhilaga et al., 2009), ameliorated Ang-II-induced aneurysm incidence without any beneficial effect on mortality rates (Vinh et al., 2008). However, we observed that MS-275, a class I HDAC inhibitor in ongoing clinical trials for oncological diseases, retards death episodes and, more interestingly, under our experimental approach, MC-1568 completely prevented mortality and reduced AAA severity. These results suggest that class IIa HDAC inhibitors constitute a promising therapeutic option for AAA patients. The beneficial effect of HDAC inhibitors was not related to either an improvement in the lipid profile or a reduction in Ang-II-induced high blood pressure. In fact, previous data showed that AAA formation in Ang-II-infused *ApoE*^−/−^ mice is independent of the increase in arterial pressure (Cassis et al., 2009; Lu et al., 2012).

VSMC apoptosis is another key feature of human AAA that contributes to the degeneration of the vascular wall (Thompson et al., 1997). Similarly, α-actin immunostaining evidenced a reduction in VSMC density in the abdominal aorta of Ang-II-infused *ApoE*^−/−^ mice that was associated with higher levels of both active caspase 3 (cleaved caspase 3), frequently used as a marker of apoptosis (Yamanouchi et al., 2012), and TUNEL staining. HDAC inhibitors attenuated these effects, suggesting that, in the vascular wall, these drugs elicit anti-apoptotic responses that could favour arterial wall stabilisation.

HDAC inhibitors also attenuated the extracellular matrix (ECM) disorganisation and degradation, which plays a pivotal role in the progressive aortic expansion observed in Ang-II-infused *ApoE*^−/−^ mice. The MMP-mediated proteolytic activity in the vessel wall is critically involved in ECM breakdown and AAA formation. MMP-2 and MMP-9 have been the most extensively studied MMPs in the context of this disease and show the strongest elastinolytic activity (Davis et al., 1998; Pyo et al., 2000; Longo et al., 2002; Luttun et al., 2004). In accordance with the lower elastin degradation and reduced collagen disorganisation exhibited by MC-1568- or MS-275-treated animals, these drugs elicited a significant reduction in vascular MMP-2 and MMP-9 expression and activity compared with Ang-II-infused *ApoE*^−/−^ mice. Indeed, epigenetic mechanisms dependent on HDAC activity are involved in the control of ECM synthesis and deposition (Pons et al., 2009), and modulate MMP expression and activity (Liu et al., 2003; Culley et al., 2013).

Humans with AAA exhibit an increase in inflammatory mediators in the vascular wall and enhanced levels of circulating cytokines and chemokines (Freestone et al., 1995; Shimizu et al., 2006). In agreement, Ang II induced a marked infiltration of macrophages, lymphocytes and neutrophils in the arterial wall from *ApoE*^−/−^ mice. Interestingly, HDAC inhibitors markedly decreased the inflammatory infiltrate in the aortic wall, accompanied by a significant reduction in the vascular expression of inflammatory markers, including COX-2, IL-1β, MCP-1 and IL-6, and an amelioration of systemic inflammation. Previous studies reported that attenuation of inflammation in AAA animal models inhibits aortic dilation, whereas an aggravated inflammatory response is associated with the progression and rupture of large aneurysms (Freestone et al., 1995; Saraff et al., 2003; Rateri et al., 2011). Interestingly, HDAC inhibitors exhibit anti-inflammatory and immunomodulatory functions regulating macrophage and lymphocyte responses under inflammatory conditions (Leoni et al., 2002; McKinsey, 2011; Licciardi et al., 2013). Based on the inhibitory effect of MC-1568 and MS-275 on local and systemic inflammation, our data suggest that HDACs could contribute to vascular inflammation in AAA through the control of the function of inflammatory cells and the consequent increase in cytokine and chemokine production.

In summary, we have shown a strong upregulation of specific HDACs in human AAA and that HDAC inhibitors limit aneurysm progression in a mouse model. Improvements in ECM remodelling, vascular apoptosis and inflammation could account for the global benefit of these drugs. Furthermore, in our preclinical model, some treatment regimens could be particularly efficient in improving survival, suggesting that these drugs might constitute a new pharmacological strategy for the treatment and stabilisation of AAA. Further research is warranted to define the molecular targets involved in their vast benefit.

## MATERIALS AND METHODS

### Human samples

Human aneurysmal samples were obtained from patients undergoing open repair for AAA at the Hospital de la Santa Creu i Sant Pau (HSCSP; Barcelona, Spain); healthy aortas were obtained from multi-organ donors as previously described (Camacho et al., 2013). Approval to use the discarded human tissue was given by the Ethics Committee of the HSCSP. Research has been carried out in accordance with the Declaration of Helsinki. Participation in the study of patients and control subjects was based upon informed consent of patients or legal representatives.

Abdominal aorta segments were obtained from all patients (*n*=22) and control subjects (*n*=14), following strict standard operating procedures and ethical guidelines. Samples of control subjects had no post-mortem evidence of abdominal aorta aneurysm, atherosclerotic plaques or other medical conditions that affect the study. Samples were rapidly collected and stored at −80°C for subsequent RNA and protein studies or processed for immunohistochemical analysis.

### Cell culture

Human aortic VSMCs were obtained from non-atherosclerotic arteries of hearts removed in transplant surgeries at the HSCSP by using a modification of the explant technique. Human VSMCs were cultured in M199 (Gibco, Carlsbad, CA) supplemented with 20% fetal calf serum (FCS), 2% human serum, 2 mmol/l L-glutamine, 100 U/ml penicillin G and 0.1 mg/ml streptomycin (Camacho et al., 2013; Orriols et al., 2014). VSMCs from at least four donors were used. Studies were performed with cells in passages 3 to 6. The research was performed in accordance with the Declaration of Helsinki and approved by the HSCSP Ethics Committee. Purity of VSMCs was confirmed by positive immunohistological staining for α-smooth muscle actin and by the negative staining for von Willebrand factor. Mycoplasma contamination was periodically analysed and excluded. For experimental procedures, subconfluent cells were arrested in medium containing 0.4% FCS for 24 h and stimulated with Ang II (10^−7^ M) for 24 h. Alternatively, VSMCs were pre-treated (1 h) with MC-1568 (5 μM; Selleck Chemicals) or MS-275 (2 μM; Selleck Chemicals). Treatments did not induce cytotoxicity as determined by the MTT assay (Roche Diagnostics, Indianapolis, IN).

### Animal handling

Animals were housed in the Animal Experimentation Unit of the Cardiovascular Research Center (CSIC-ICCC, Barcelona, Spain) in a controlled, specific pathogen-free environment under standard light-dark cycle (12 h light/dark cycle) and temperature (21±1°C) conditions, and were fed *ad libitum* with a standard commercial diet (Harlan Iberica SL, Barcelona, Spain). All animal handling procedures were performed in compliance with the principles and guidelines established by the Spanish Policy for Animal Protection RD53/2013, which meets the European Union Directive 2010/63/UE on the protection of animals used for experimental and other scientific purposes, and all procedures were reviewed and approved by the Ethical Committee at the Centro de Investigación Cardiovascular as stated in Law 5/1995, 21 June, passed by the Generalitat de Catalunya.

The apolipoprotein-E-deficient (*ApoE*^−/−^) mouse infused with angiotensin II (Ang II) was used as a model of AAA as previously described (Daugherty et al., 2000). 10-week-old male *ApoE*^−/−^ mice (*ApoE*^−/−^; B6.129P2-*Apoe^tm1Unc^*/J) were obtained from Charles River UK Ltd (Kent, UK). Mice were acclimated 1 week prior to the study. Ang II (1000 ng kg^−1^ body weight min^−1^; Sigma-Aldrich, St Louis, MO) was infused via osmotic minipumps (model 1004, Alzet, DURECT Corporation, CA) implanted subcutaneously into the interscapular space of isoflurane-anaesthetised mice for 28 days. Animals were randomly distributed in five experimental groups: untreated Ang-II-infused mice (*n*=20); two groups of Ang-II-infused mice that received an intraperitoneal injection of histone deacetylases (HDACs) inhibitors (Selleck Chemicals, Houston, TX) [MC-1568 (a class-IIa-HDAC-specific inhibitor; 50 mg kg^−1^ body weight) or MS-275 (a class-I-HDAC-specific inhibitor; 20 mg kg^−1^ body weight) (*n*=13 each group)] 3 days a week; a group of Ang-II-infused *ApoE*^−/−^ mice receiving the broad-spectrum metalloproteinase (MMP) inhibitor doxycycline (doxycycline hyclate, Sigma-Aldrich; 30 mg kg^−1^ body weight day^−1^), which has previously been demonstrated to inhibit AAA formation in this model (Manning et al., 2003) (administered in the drinking water; *n*=6); and a control group of *ApoE*^−/−^ mice infused with saline (*n*=18). Doses of HDAC inhibitors were chosen according to previous *in vivo* studies (Nebbioso et al., 2009; Spallotta et al., 2013; Dalgard et al., 2008), and treatments started 3 days before minipump implantation.

For the implantation of osmotic minipumps, mice were anaesthetised with isofluorane (2%), which has a rapid effect on animals. Anaesthetic depth was confirmed by loss of blink reflex and/or lack of response to tail pinch. The procedure takes about 15 min/mouse. Recovery after surgical procedures was carried out using aseptic techniques in a dedicated approved surgical area. Antibiotics (penicillin, 450,000 u kg^−1^, intramuscular) and analgesics (buprenorphine 0.05 mg kg^−1^, subcutaneous) were given immediately after surgery to prevent infection and discomfort. The animals were kept warm in a heating pad until awake after surgery, and observed carefully by the investigators throughout the post-surgery period. At the end of the experimental procedures, mice were euthanised via isofluorane overdose and the aortas were immediately harvested, examined for the presence of an AAA and appropriately processed for further studies.

### Non-invasive measurement of systolic blood pressure

Systolic blood pressure (SBP) and mean arterial pressure (MAP) were non-invasively measured in conscious mice prior to and following treatment using the tail-cuff plethysmography method (Panlab, Harvard Apparatus). Mice were trained for tail-cuff measurements over a period of 1 week. Blood pressure measurements were performed at the same time (between 9 a.m. and 11 a.m.) in order to avoid the influence of the circadian cycle. Mean blood pressure values were taken from ten consecutive measurements (Orriols et al., 2014).

### Basic measurements of ultrasound recording for abdominal aortas

Mice were anaesthetised with 1.5% isofluorane inhalation and were lightly secured in the supine position to a warming platform. After shaving the precordium, an abdominal echography was performed using a Vevo 2100 ultrasound with a 30 MHz transducer applied to the abdominal wall to record abdominal aorta (VisualSonics, Toronto, Canada). Abdominal aortas with external diameters ≥1.5 mm were considered as an aneurysm. All primary measurements were made from images captured on cine loops of 100 frames at the time of the study using the software provided by the echography machine.

The severity of the aneurysm was based on a 4-point grading scale previously described in detail (Manning et al., 2003): type 0, no aneurysm; type I, dilated lumen in the suprarenal region of the aorta with no thrombus; type II, remodelled tissue in the suprarenal region that frequently contained thrombus; type III, a pronounced bulbous form of type II that contained thrombus, and type IV, a form in which there are multiple AAAs containing thrombus.

### Total mRNA and protein isolation from tissues

The RNeasy Mini Kit (Qiagen, Venlo, Netherlands) was used to isolate total RNA from human AAA samples following the manufacturer's recommendation. Protein lysates from human AAA were prepared in a RIPA buffer [150 mM NaCl, 1% (v/v) Triton X-100, 0.5% (w/v) sodium deoxycholate, 0.1% (w/v) SDS, 2 mM EDTA, 50 mM Tris-HCl pH 8] by using a tissue homogenizer and following a standard protocol.

In the case of animal studies, after removal of adventitial fat from mouse aortas, the aneurysm was separated, snap-frozen in liquid nitrogen and stored at −80°C. Total RNA and protein isolation from mouse abdominal aorta was performed using the Tripure reagent (Roche Diagnostics, Indianapolis, IN) following the manufacturer's instructions. RNA integrity was determined by electrophoresis in agarose gels and was quantified by a NanoDrop 1000 Spectrophotometer (Thermo Scientific).

### Real-time PCR

DNase-I-treated total RNA (1 μg) was reverse transcribed into cDNA using the High Capacity cDNA Archive Kit (Applied Biosystems, Foster City, CA) with random hexamers. Quantification of mRNA levels was performed by real-time PCR using an ABI PRISM 7900HT sequence detection system (Applied Biosystems) and specific primers and probes provided by the Assay-on-Demand system (Applied Biosystems) for human *HDAC1* (Hs02621185_s1), *HDAC2* (Hs00231032_m1), *HDAC3* (Hs00187320_m1), *HDAC8* (Hs00218503_m1), *HDAC4* (Hs01041638_m1), *HDAC5* (Hs00608366_m1), *HDAC7* (Hs00248789_m1), *MMP2* (Hs00234422_m1), interleukin 1β (IL1β; Hs00174097_m1), chemokine (C-C motif) ligand 2 (*CCL-2* or *MCP-1*; Hs00234140_m1) and prostaglandin-endoperoxide synthase 2 (*PTGS2* or *COX-2*; Hs00153133_m1). 18S rRNA (4319413E) was used as an endogenous control for human samples. To analyse mRNA levels in mouse tissues, we used TaqMan fluorescent real-time PCR primers and probes, provided by Applied Biosystems or Integrated DNA technologies as follows: *IL6* (Mm00446191_m1), *IL-1β* (Mm00434228_m1), *MCP-1* (Mm00441242_m1), *COX-2* (Mm00478374_m1), *HDAC1* (MmPT.58.14183463), *HDAC2* (MmPT.58.13367660), *HDAC3* (MmPT.58.13259320), *HDAC4* (MmPT.58.17110912), *HDAC5* (MmPT.58.16075245), *HDAC7* (MmPT.58.13463976), *HDAC8* (Mm01224980_m1), *MMP-9* (MmPT.58.10100097), *MMP-2* (MmPT.58.9606100), *EMR-1* (MmPT.56a.11087779) and Elane (elastase, neutrophil expressed; Mm00469310_m1). As endogenous controls for mouse samples, glyceraldehyde 3-phosphate dehydrogenase (*GAPDH*; Mm99999915_g1) and 18S rRNA (4319413E) were used. Quantitative RT-PCR was carried out in an ABI PRISM 7900HT Sequence Detection System (Applied Biosystems) using the following conditions: 2 min at 50°C, 10 min at 95°C followed by 40 cycles of 15 s at 95°C and 1 min at 60°C. Relative mRNA levels were determined using the 2^−ΔΔCt^ method.

### Western blot

Tissue lysates were separated by SDS-PAGE and transferred to 0.45 μm polyvinylidene difluoride membranes (Immobilon, Millipore, Merck KGaA, Darmstadt, Germany). Blots were incubated with antibodies directed against HDAC1 (NBP1-67590), HDAC2 (NB100-91802) and HDAC4 (NBP1-67592) purchased from Novus Biologicals (R&D Systems Europe Ltd, Abingdon, UK); antibodies against HDAC7 (ab166911) and histone H3 acetyl K18 (Ac-H3K18; ab1191) were obtained from Abcam (Cambridge, UK). All were used at 1:1000 dilution. Equal loading of protein in each lane was verified by β-actin (A5441, Sigma-Aldrich).

### Immunohistochemistry, immunofluorescence and histology

Aortic samples were fixed in 4% paraformaldehyde/0.1 M phosphate buffered saline (PBS; pH 7.4) for 24 h and embedded in paraffin. Aortic sections from human and mouse samples (5 μm) were deparaffinised in xylene, rehydrated in graded ethanol, and treated with 0.3% hydrogen peroxide for 30 min to block peroxidase activity. Then, samples were blocked with 10% of normal serum and incubated with antibodies against LAMP-2/Mac-3 (sc-19991, 1:250, Santa Cruz Biotechnology Inc., Europe), CD3 (A0452, 1:100, Dako, Agilent Technologies Co., Hamburg, Germany), MCP-1 (sc-1785, 1:100, Santa Cruz Biotechnology Inc.), Elane (M0752, Dako, Agilent Technologies Co., Hamburg, Germany), cleaved-caspase 3 (9661, 1:100, Cell Signaling, Boston, MA), HDACs and Ac-H3K18 overnight at 4°C (both at 1:100). After washing, samples were incubated for 1 h with a biotinylated secondary antibody (Vector Laboratories, Peterborough, UK). After rinsing three times in PBS, standard Vectastain avidin-biotin peroxidase complex (ABC; Vector Laboratories) was applied, and the slides were incubated for 30 min. Colour was developed using 3,3′-diaminobenzidine (DAB) and sections were counterstained with haematoxylin before dehydration, clearing and mounting. Negative controls, in which the primary antibody was omitted, were included to test for non-specific binding.

For immunofluorescence studies, mouse abdominal aorta sections were blocked in PBS containing 5% BSA and 0.1% Triton X-100, and then sections were incubated with a polyclonal antibody against MMP-9 (sc-10737, 1:100, Santa Cruz Biotechnology), a rabbit monoclonal antibody against MMP-2 (ab51125, 1:100, Abcam), or a rabbit polyclonal antibody against α-actin (ab5694, Abcam), α-actin (M0851, 1:200, Dako, Agilent Technologies Co., Hamburg, Germany), CD3 and HDACs overnight at 4°C. After washing, rings were incubated with an anti-rabbit IgG conjugated to TRITC or FITC probes (Dako) for 1 h at room temperature (RT). After washing, slides were mounted with SlowFade Gold antifade reagent with DAPI (Molecular Probes, Life Technologies, Madrid, Spain) and immunofluorescent signals were viewed using a Nikon Eclipse 55i fluorescence microscope. Results were quantified and expressed as percentage of positive area versus total area and as positive cell number in independent sections of AAA for CD3, Mac-3, MCP-1, neutrophil elastase and cleaved-caspase 3. Negative controls, in which the primary antibody was omitted, were included to test for non-specific binding. The histological characterisation of aortic samples was performed by Masson's trichrome staining. Furthermore, to visualise elastic fibre integrity, arterial sections were stained with orcein using a commercial kit (Casa Álvarez, Madrid, Spain).

### Double-fluorescence immunostaining

For antigen colocalization studies, double-fluorescence immunostaining was performed using a sequential method. After deparaffinization, antigen retrieval and permeabilization with PBS-0.1% Triton X-100, sections were blocked with PBS containing 5% albumin (4°C overnight). Incubation with rabbit polyclonal antibodies against HDAC1, HDAC2 and HDAC4, and those against smooth muscle α-actin or CD3 (1:100), were then sequentially applied. After washing the secondary antibodies, donkey anti-mouse IgG conjugated to Alexa Fluor 542, and chicken anti-rabbit IgG conjugated to Alexa  Fluor 488 (Molecular Probes, Life Technologies) were applied for 1 h at RT. Negative controls, in which the primary antibody was omitted, were also included. Samples were then mounted with ProLong Gold antifade reagent with DAPI (Molecular Probes, Life Technologies Co., Eugene, OR). Images were obtained using an SP5 Leica confocal microscope.

### Gelatin zymography

The relative enzymatic activities of MMP-9 and MMP-2 in murine abdominal aorta lysates were measured by zymography. Protein lysates were prepared in RIPA buffer supplemented with complete protease inhibitor cocktail (Roche). Proteins (20 μg) were resolved by 10% SDS-polyacrylamide gels copolymerised with 1 mg ml^−1^ of porcine skin type A gelatin (Sigma-Aldrich) as a substrate for MMP enzymatic activity, and run at 4°C for 4-6 h. After electrophoresis, the gels were rinsed twice for 30 min at RT in 2.5% Triton X-100 (Sigma), and then incubated in substrate buffer [50 mM Tris-HCl, 10 mM CaCl_2_ (Merck), 0.02% (w/v) N_3_Na (Fluka), pH 8] for 18-20 h at 37°C. Gels were dyed with one tablet of PhastGel™ Blue R (GE Healthcare) in 10% acetic acid. Areas of gelatinolytic activity appeared as clear bands on a blue background where the protease has digested the substrate. Gels were finally scanned with a GS-800 calibrated imaging densitometer (Bio-Rad) and quantitative densitometric analysis of digested bands was performed using Quantity-One software (Bio-Rad) (Rodríguez-Calvo et al., 2015).

### Enzyme-linked immunosorbent assay (ELISA)

IL-6 and IL-2 concentrations in plasma were determined using the Quansys Q-plex mouse cytokine system (Quansys Biosciences, UT).

### Determination of the lipid profile in plasma

Lipids were quantified with a colorimetric assay in plasma from all groups of mice by using specific reagents for triglycerides (Gernon, GN90125; RAL Técnica para el Laboratorio S.A., Barcelona, Spain), total cholesterol (Gernon, GN20125) and high-density lipoprotein (HDL) cholesterol (Gernon, GN20525), and a multicuvette rack reader (Clima MC-15, RAL Técnica para el Laboratorio S.A., Barcelona, Spain) following the manufacturers’ instructions. HDL was quantified by the phosphotungstic acid method using a COBAS 6000 analyzer (Roche Diagnostics, USA) as described (Ribas et al., 2004).

### TUNEL assay

Recombinant terminal deoxynucleotidyl transferase (rTdT)-mediated nick-end labelling (TUNEL) was performed using the Dead End Fluorometric Tunel System (Promega) according to the manufacturer's guidelines. Four-micrometer paraffin sections were deparaffinised, fixed in methanol-free paraformaldehyde before and after proteinase K treatment at 20 μg/ml for 8-10 min at RT. The sections were incubated with the nucleotide mixture (which included fluorescein-tagged dUTP) and rTdT enzyme for 1 h at 37°C. The slides were mounted using SlowFade Gold antifade reagent with DAPI (Molecular Probes, Invitrogen) and immunofluorescent signals were viewed using a fluorescence microscope (Nikon Eclipse 55i).

### Statistical analysis

Data were expressed as mean±s.e.m. and were analysed using the GraphPad Prism 4.0 software (GraphPad, USA). Human and animal population data were expressed as mean±s.e.m. Values of *P*≤0.05 were considered significant. Differences between specified groups were analysed using the Student's *t*-test (two-tailed) comparing two groups. One-way ANOVA and the Bonferroni test were used to determine the statistical significance of the observed differences between the studied groups. When normality failed, we used the Mann–Whitney rank sum test to compare two groups and Kruskal–Wallis one-way ANOVA on ranks for multiple comparisons (Dunn's method). Differences in the trends for mortality between groups were determined using Kaplan–Meier test and differences in the percentage of incidence of AAA were analysed by Chi square test (χ^2^).
